# Supplementary material for: Breakage of the oligomeric CaMKII hub by the regulatory segment of the kinase
Source: eLife. 2020 Sep 9;9:e57784. doi: 10.7554/eLife.57784 (PMC7538161; doi:10.7554/eLife.57784)
Supplement: Transparent reporting form [file elife-57784-transrepform.pdf]

eLIFE
Westbrook Centre, Milton Road
Cambridge CB4 1YG
UK
P 01223 855340
W elifesciences.org
T @elife

eLife's transparent reporting form

We encourage authors to provide detailed information *within their submission* to facilitate the interpretation and replication of experiments. Authors can upload supporting documentation to indicate the use of appropriate reporting guidelines for health-related research (see EQUATOR Network), life science research (see the BioSharing Information Resource), or the ARRIVE guidelines for reporting work involving animal research. Where applicable, authors should refer to any relevant reporting standards documents in this form.

If you have any questions, please consult our Journal Policies and/or contact us: editorial@elifesciences.org.

Sample-size estimation

- You should state whether an appropriate sample size was computed when the study was being designed
- You should state the statistical method of sample size computation and any required assumptions
- If no explicit power analysis was used, you should describe how you decided what sample (replicate) size (number) to use

Please outline where this information can be found within the submission (e.g., sections or figure legends), or explain why this information doesn't apply to your submission:

Details of sample size and the assumptions made for analyses are provided in the Materials and Methods section.

Replicates

- You should report how often each experiment was performed
- You should include a definition of biological versus technical replication
- The data obtained should be provided and sufficient information should be provided to indicate the number of independent biological and/or technical replicates
- If you encountered any outliers, you should describe how these were handled
- Criteria for exclusion/inclusion of data should be clearly stated
- High-throughput sequence data should be uploaded before submission, with a private link for reviewers provided (these are available from both GEO and ArrayExpress)

Please outline where this information can be found within the submission (e.g., sections or figure legends), or explain why this information doesn't apply to your submission:

Mass spectrometry experiments:
Each experiment has been repeated with three different sub-micron emitters.

Single-molecule experiments:
1. Each experiment reported in the article was replicated at least 3 times (as mentioned in Materials and Methods).

2. The biological replication involves the use of different batches of HEK293 cells, different batches of DNA used for overexpression and experimental samples prepared on different days. For technical replication, the flow chambers for each experiment were freshly prepared on the day of the experiment. In addition, for each sample, we acquired single-molecule images over 60 distinct frames amounting to 3000-20000 single particles. These were analyzed to generate intensity distribution plots (see Materials and Methods for details of sample preparation, data collection and analyses).

3. The cut-offs applied, exclusions of data and all assumptions underlying data analyses are discussed in the Materials and Methods section (see sub-section - Single-molecule Total Internal Reflection Fluorescence (TIRF) microscopy AND Analyses of single-molecule TIRF data).

Statistical reporting

- Statistical analysis methods should be described and justified
- Raw data should be presented in figures whenever informative to do so (typically when N per group is less than 10)
- For each experiment, you should identify the statistical tests used, exact values of N, definitions of center, methods of multiple test correction, and dispersion and precision measures (e.g., mean, median, SD, SEM, confidence intervals; and, for the major substantive results, a measure of effect size (e.g., Pearson's r, Cohen's d)
- Report exact p-values wherever possible alongside the summary statistics and 95% confidence intervals. These should be reported for all key questions and not only when the p-value is less than 0.05.

Please outline where this information can be found within the submission (e.g., sections or figure legends), or explain why this information doesn't apply to your submission:

All figures in the single-molecule analysis experiments are typically the result of analyses of 3000-20000 single molecules of CaMKII. The standard deviations are reported as error bars on the figures reporting colocalization.

The distributions are histograms generated from the intensity values of these particles (bin width 2165) and the detailed analyses, normalization and data representation are provided in the Materials and Methods section.

(For large datasets, or papers with a very large number of statistical tests, you may upload a single table file with tests, Ns, etc., with reference to sections in the manuscript.)

Group allocation

- Indicate how samples were allocated into experimental groups (in the case of clinical studies, please specify allocation to treatment method); if randomization was used, please also state if restricted randomization was applied
- Indicate if masking was used during group allocation, data collection and/or data analysis

Please outline where this information can be found within the submission (e.g., sections or figure legends), or explain why this information doesn't apply to your submission:

No experiments in this submission required group allocation.

Additional data files ("source data")

- We encourage you to upload relevant additional data files, such as numerical data that are represented as a graph in a figure, or as a summary table
- Where provided, these should be in the most useful format, and they can be uploaded as "Source data" files linked to a main figure or table
- Include model definition files including the full list of parameters used
- Include code used for data analysis (e.g., R, MatLab)
- Avoid stating that data files are "available upon request"

Please indicate the figures or tables for which source data files have been provided:

eLife Sciences Publications, Ltd is a limited liability non-profit non-stock corporation incorporated in the State of Delaware, USA, with company number 5030732, and is registered in the UK with company number FC030576 and branch number BR015634 at the address Westbrook Centre, Milton Road Cambridge CB4 1YG, UK | March 2019

1. Raw data for all figures that plot numerical data for the single-molecule experiments and molecular dynamics simulations are provided in files called “Figure X-Source data Y”

2. The details of the code used for single-molecule data analyses are provided in the Materials and Methods section (see sub-section - Analyses of single-molecule TIRF data), and the code itself is included.
